# Successful Management of Life-threatening Pelvic Hemorrhage From Acquired Factor V Deficiency With immunosuppressive Therapy

**DOI:** 10.7759/cureus.9972

**Published:** 2020-08-23

**Authors:** Alexander H Wu, Anusha Manje Gowda, Sharon Peng, Supreet Kaur, Michael Maroules

**Affiliations:** 1 Internal Medicine, St. Joseph's University Medical Center, Paterson, USA; 2 Hematology/Oncology, St. Joseph's University Medical Center, Paterson, USA

**Keywords:** acquired factor v deficiency, immunosuppressive therapy, bleeding diathesis

## Abstract

Acquired Factor V deficiency is a rare and challenging condition to treat. It has been associated with major surgeries, antibiotics, blood transfusions, infections, autoimmune disorders, malignancy and exposure to bovine thrombin. The clinical presentation can be heterogeneous and can manifest as asymptomatic laboratory abnormalities to fatal hemorrhage with mortality rates around 15-20% . We report a case of acquired factor V deficiency in which the patient developed a life-threatening bleeding coagulopathy with elevated prothrombin time, activated partial thromboplastin time and factor V inhibitor titers following multiple surgical procedures that were performed after a motor vehicle accident. The patient was successfully treated with immunosuppressive therapy including steroids and cyclophosphamide resulting in the complete elimination of inhibitor levels.

## Introduction

Acquired Factor V deficiency is a rare and challenging condition to treat. Its presentation can be heterogeneous, ranging from an asymptomatic clinical state with abnormal coagulation studies to life-threatening bleeding. If not recognized and treated promptly, it can be fatal. Here we describe a patient who developed acquired factor V deficiency resulting in life-threatening hemorrhage after undergoing multiple surgeries following a motor vehicle collision, requiring immunosuppressive therapy for the elimination of inhibitors.

## Case presentation

A 63-year-old male of Hispanic descent with a past medical history of Diabetes Mellitus presented to our hospital after a motor vehicle accident that resulted in multiple fractures including rib, femoral and pelvic fractures. He had no personal or familial history of bleeding disorders. He had undergone minor surgeries and dental extractions in the past without any occurrence of excessive bleeding or requirement of blood transfusions. Whole-body imaging done as per the trauma code protocol revealed multiple rib fractures, dislocation of the right femur, and a right acetabular fracture extending to the right inferior pubic ramus. The patient developed hemorrhagic shock requiring multiple blood products including packed red blood cells, fresh frozen plasma, cryoprecipitate. He underwent multiple orthopedic procedures such as open reduction internal fixation (ORIF) of the bilateral sacral iliac joints, pubic symphysis, and right acetabulum. He also underwent arterial embolization of the left inferior branch of the left internal iliac artery for right-sided lower pelvic hematoma. On day sixteen of hospital admission, the patient developed worsening right groin pain and swelling. Computed Tomography of the pelvis revealed an expanding 13 x 10 x 10-centimeter hematoma; evacuation of the hematoma was performed and a wound vac was placed. He continued to have persistent bloody output from the wound vac, requiring daily packed red cell transfusions. On Day 17, the patient’s Prothrombin Time (PT) and Activated Partial Thromboplastin Time (aPTT) were abnormally elevated at 66.0 s and >140 seconds respectively. The PT and aPTT were normal on admission at 14.5 seconds and 23.4 seconds respectively. The laboratory findings are shown in Table 1. 

**Table 1:** Laboratory Data PT- Prothrombin Time aPTT- activated partial thromboplastin time

Test Name	Results	Reference Range	Units
White cell count	13.5	4.5-11.0	K/mm3
Hemoglobin	7.0	13.5-17.5	g/dL
Hematocrit	20.2	41.0-53.0	%
Platelet count	556	140-440	K/mm3
Red cell distribution width (RDW)	20.2	0.5-16.5	%
International Normalized Ratio (INR)	7.5		
Activated partial thromboplastin time (aPTT)	>140.0	21.3-35.1	Seconds
Prothrombin time (PT)	66.0	12.1-14.9	Seconds
Fibrinogen	582	185-503	mg/dL
Fibrin degradation products (FDP)	>20	<5	ug/mL
D Dimer	4.69	<0.50	ug/mL
Clotting factor 5	6	58-123	%
Clotting factor 10	102	74-132	%
Clotting factor 8	523	49-126	%
PT Mixing study	56.1	12.2-14.9	seconds
PT 1:1 dilution	48.0		Seconds
aPTT mixing study	>140	21.3-35.1	seconds
aPTT1:1 dilution	106.4		Seconds
aPTT 1:1 60 min	>140		Seconds
aPTT 4:1 dilution	126.6		Seconds
Factor V inhibitor	3.8		

Despite receiving fresh frozen plasma, packed red cells, platelets and cryoprecipitate, there was no improvement in his hemodynamics or coagulation studies. A mixing study failed to correct the abnormal aPTT (both 0 and 1-hour incubation) suggesting the presence of an inhibitor and the Factor V activity was decreased at 6%. A standard Bethesda assay identified a Factor V inhibitor titer of 3.8 Bethesda units. The patient was treated with a combination of daily intravenous immunoglobulin (IVIG) for three doses and methylprednisolone at 1mg/kg/day with a lack of clinical improvement. Cyclophosphamide was started subsequently at a dose of 100 mg oral daily, following which, the patient’s bleeding symptoms eventually ceased and the PT and aPTT normalized within 10 days of initiating immunosuppressive treatment. Factor V inhibitor titers were undetectable and the activity was > 70% at three weeks after immunosuppressive therapy was initiated. Cyclophosphamide was eventually tapered off and the patient continued to clinically improve. Coagulation studies and hemoglobin remained stable for several weeks after stopping treatment. 

## Discussion

Factor V (FV) plays an essential role in the coagulation cascade; it is a cofactor necessary for the prothrombinase complex to activate prothrombin into thrombin [1, 2]. Congenital FV deficiency is rare; it is estimated that 1 in 1, 000,000 people are affected [ 1 ]. Even rarer are acquired FV inhibitors, which make up a small percentage of those with FV deficiency [1, 3]. Although FV inhibitors may occur at all ages, most cases occur at around 65 years of age and predominantly in males [4, 5]. A little more than 150 cases of acquired FV inhibitors have been reported [5]. The clinical presentation can be heterogeneous and can manifest as asymptomatic laboratory abnormalities to fatal hemorrhage with mortality rates around 15-20% [4, 5]. 

According to multiple reviews, acquired factor V inhibitors have been described in association with several conditions such as autoimmune diseases, antibiotics (such as β-lactams, aminoglycosides, cephalosporins), pregnancy, surgical procedures, blood transfusion, infections, malignancy, amyloidosis, and exposure to bovine thrombin glue which is used as a hemostatic agent in surgical and orthopedic procedures [4, 5]. It is reported that most cases of acquired FV inhibitors are seen after exposure to bovine thrombin. Antibodies can develop against bovine factor V found in small amounts in the glue resulting in a cross-reaction with human factor V [4]. FV inhibitors have also been described after exposure to human thrombin, which is very rare [2]. 

Asymptomatic patients generally do not require treatment [4]. The inhibitors are usually transient, especially in cases where inhibitors are induced by bovine thrombin [5]. However, in cases where the patient has a life-threatening bleed, such as in our case, the goal is to reduce or eliminate the inhibitors [6, 5]. There is no standard therapy for acquired factor inhibitors; however, there are different proposed treatment options with multiple systematic reviews demonstrating varying success rates [4, 5, 6]. Evidence regarding the use of fresh-frozen plasma and prothrombin complex concentrates has shown lower success rates [4]. Platelet transfusions seem to be more promising with a success rate of 71% [5]. The proposed mechanism is that Factor V is present within platelet α-granules and is activated and degranulated at the bleeding site, thus contributing to hemostasis. [5, 7]. Intravenous immunoglobulin infusion and plasmapheresis have been shown to be effective in reducing inhibitors rapidly with a success rate of 60% and 53% respectively [4]. Immunosuppressant therapy with steroids alone or in conjunction with cyclophosphamide, rituximab, or other agents has shown a success rate of 63% in suppressing the antibodies and a remission rate of 76% [5]. It has also been reported that the level of antibodies did not correlate with the extent of bleeding or the clinical picture [6] 

Our patient underwent multiple surgeries and received antibiotics for prolonged duration in addition to multiple blood transfusions; there might have been a possible exposure to thrombin glue during the surgical procedures. One or more of these risk factors may have played a potential role in the development of FV inhibitors leading to life-threatening bleeding. He did not respond to platelet transfusions, high dose steroids, and IVIG. However, with the initiation of cyclophosphamide, the patient’s symptoms markedly improved and the PT and aPTT normalized within 10 days of immunosuppressive therapy.

## Conclusions

The precise pathophysiology of factor V inhibitor formation has not been elucidated and the rarity of the condition and heterogeneity of clinical presentation makes a prompt diagnosis challenging. Physicians and surgeons should keep a high index of suspicion in patients who develop coagulopathy postoperatively in order to allow for timely recognition and appropriate intervention. In this article, we have also attempted to shed light on the various treatment modalities that have been successful so far in treating this rare and difficult condition.

## References

[1] Huang JN, Koerper MA (2008 Nov). Factor V deficiency: a concise review. Haemophilia.

[2] Donohoe K, Levine R (2015). Acquired factor V inhibitor after exposure to topical human thrombin related to an otorhinolaryngological procedure. J Thromb Haemost.

[3] Patel MD, Hajdenberg J (2016). Successful treatment of chronic recurrent life-threatening bleeding due to an acquired factor V Inhibitor with rituximab and steroids. Haemophilia.

[4] Knöbl P, Lechner K (1998). Acquired factor V inhibitors. Baillieres Clin Haematol.

[5] Franchini M, Lippi G (2011). Acquired factor V inhibitors: a systematic review. J Thromb Thrombolysis.

[6] Ang AL, Kuperan P, Ng CH, Ng HJ (2009). Acquired factor V inhibitor: a problem-based systematic review. Thromb Haemost.

[7] Chediak J, Ashenhurst JB, Garlick I, Desser RK (1980). Successful management of bleeding in a patient with factor V inhibitor by platelet transfusions. Blood.

